# Correspondence

**Published:** 1915-12

**Authors:** F. Klein, Chas. Walker


### CORRESPONDENCE.

This department is intended for the presentation of news and views not coming within the scope of other departments, and particularly to afford opportunity for discussion and criticism of views elsewhere expressed.

---

327 West 86th Street,
N. Y. City, Nov. 13th, 1915.

Dr. A. L. Benedict,
Editor of the Buffalo Medical Journal,
Buffalo, N. Y.

Dear Dr. Benedict:—

In the last number of the Buffalo Medical Journal we have read the abstract from our previous published Pregnancy Test, which you have published.

Allow us on this occasion to send you a reprint copy of same, besides giving further details regarding the Test and chemicals used.

The "**Iron Reagent,**" which is the main Test solution forms a Ferrate, the so-called Eisensaure (Fe O3) which is a higher oxygen iron compound, than Ferric oxyd (or Hydroxyd. Fe2 O3.) Former compound is found in the spleen, liver and blood according to our research, the addition of glycerine as stated prevents coagulation of the formed colloidal Eisensaure and in connection with strong hydrochloric acid 37-38% c. p. is the main feature of the reagent.

We also wish to remark that the copper-sod. hydrate reagent as first check Test is preferable for several reasons, it is just as accurate as the Bensidinseleno sol. and easier to manipulate and to obtain the chemicals necessary.

Hoping that this information may be welcomed and any further called for we will gladly accommodate.

Yours very respectfully,

Dr. F. KLEIN.

Dr. CHAS. WALKER.

